# Factors contributing to fidelity in a pilot trial of individualized resistant starches for pediatric inflammatory bowel disease: a fidelity study protocol

**DOI:** 10.1186/s40814-021-00815-1

**Published:** 2021-03-19

**Authors:** Gisell Castillo, David R. Mack, Manoj M. Lalu, Ruth Singleton, Dean A. Fergusson, Alain Stintzi, Megan Harrison, Justin Presseau

**Affiliations:** 1 Clinical Epidemiology Program, Ottawa Hospital Research Institute, 501 Smyth Road, Ottawa, Ontario K1H 8L6 Canada; 2 Children’s Hospital of Eastern Ontario Inflammatory Bowel Disease Centre, Ottawa, Ontario Canada; 3 Department of Pediatrics, University of Ottawa, Ottawa, Ontario Canada; 4 Children’s Hospital of Eastern Ontario Research Institute, Ottawa, Ontario Canada; 5 Department of Anesthesiology and Pain Medicine, The Ottawa Hospital, Ottawa, Canada; 6 Department of Cellular and Molecular Medicine, University of Ottawa, Ottawa, Canada; 7 School of Epidemiology and Public Health, University of Ottawa, Ottawa, Ontario Canada; 8 Department of Biochemistry, Microbiology and Immunology, University of Ottawa, Ottawa, Ontario Canada; 9 Department of Psychology, University of Ottawa, Ottawa, Ontario Canada

**Keywords:** Fidelity, Inflammatory bowel disease, Resistant starches, Personal projects analysis, Qualitative research

## Abstract

**Background:**

The consumption of resistant starches is a promising adjuvant therapy for patients with inflammatory bowel disease. Rigorous evaluation of resistant starches in this setting depends on the intervention being delivered, received, and enacted as intended, that is, with fidelity. As part of a planned pilot trial, participants will be randomized to ingest resistant starches or a placebo. They will also be asked to collect stool samples and keep symptom and dose diaries to inform trial outcomes.

We aim to identify potential factors impacting fidelity to the receipt and enactment of trial intervention and data collection activities from the perspective of patients and caregivers in the trial. Identifying fidelity barriers and enablers at the pilot trial phase of a clinical intervention may help to determine optimization processes when expanding to multiple sites in future trials.

**Methods:**

We will conduct 15-30 semi-structured interviews with pilot trial participants (aged 8-17) and their caregivers. Trial participants will be approached for interviews approximately 6 months after the start of their trial participation. Personal projects analysis, a tool for understanding how individuals manage competing demands in their daily lives, will guide an in-depth exploration of how trial participants engage in activities related to intervention and data collection fidelity (ingesting resistant starches or placebo, collecting stool samples, keeping a symptom and dose diary) amidst the complexities of daily living.

**Discussion:**

The present study will seek to explore and demonstrate how theory-informed fidelity assessments can be conducted alongside pilot trials to inform future multisite trials. Study results will clarify what factors may affect fidelity to trial intervention and data collection activities. Results may suggest what to modify to optimize the design and conduct, and ensure the integrity, of future multisite trials. Conducting process evaluations alongside clinical trials has the potential to improve our understanding of trial participant experiences. Results will provide a better understanding of how trial participants manage to engage in necessary trial activities along with other priorities.

**Supplementary Information:**

The online version contains supplementary material available at 10.1186/s40814-021-00815-1.

## Background

The importance and value of pilot and feasibility studies are increasingly recognized as core research activities prior to launching resource-intensive clinical trials [1, 2]. When trials involve several participating sites, the differences in resources, processes, and expertise between sites introduce a variety of implementation and fidelity challenges. Unaddressed, such fidelity challenges can impact the success of the trial [3]. Conducting process evaluations alongside pilot trials can clarify what works and suggest ways of optimizing the design and conduct of future larger-scale or multisite trials [1, 4]. Herein, we describe our proposed methods for conducting a fidelity-focused process evaluation alongside a pilot randomized controlled trial of resistant starches as an adjuvant therapy for pediatric inflammatory bowel disease.

### Study context: pediatric inflammatory bowel disease

The incidence of pediatric inflammatory bowel disease (IBD) in Canada is among the highest in the world and continues to rise, with hundreds of new cases identified every year [5–7]. The two most common subtypes of IBD, Crohn’s disease (CD) and ulcerative colitis (UC), are characterized by acute and chronic intestinal inflammation that can lead to intestinal scarring, fistulas, abscesses, rectal bleeding, cancer, and the need for surgery (e.g., colectomies and ostomies) [6, 7]. Children diagnosed with IBD may experience a number of different symptoms including pain, growth impairments, diarrhea, bloody stools, weight changes, fatigue, as well as difficulties with psychosocial functioning and school performance compared to children who do not live with IBD [6, 8, 9]. The challenges of living with IBD often extend to caregivers who may experience socioeconomic hardships and elevated levels of stress [6, 10]. While there is no known cure for IBD, researchers continue to identify genetic, immunologic, environmental, and intestinal dysbiosis risk factors that predispose individuals to IBD and impact disease outcomes [6, 11–13].

A pilot trial is currently underway (ClinicalTrials.gov Identifier: NCT04522271) that seeks to assess how ingesting individualized food-derived resistant starches may enhance the intestinal microbiome functioning and anti-inflammatory properties, and whether there are any associated changes in clinical outcomes (e.g., severity of inflammation). Pediatric patients, with a new diagnosis of either ulcerative colitis or Crohn’s disease, will be invited to participate in this pilot trial. Those that consent will be randomized to receive an individually optimized resistant starch food product or a placebo. Participants will also be asked to (1) take the resistant starches/placebo daily for the 6-month intervention period, (2) collect stool samples (3-5 samples total over the year of the study), (3) keep a symptom, dose, and adverse events diary, and (4) adhere to standard care medication.

### Assessing fidelity barriers

Trial findings and their interpretation depend on whether participants complete trial-related activities as instructed, agreed upon, and expected, that is, with fidelity [3, 14]. For example, ensuring that participants take resistant starches as prescribed, properly collect stool samples to ensure the resulting samples can be used for trial outcome data, record symptoms, adverse events, and missed doses in a diary, and adhere to standard care regimens is critical to preserve a trial’s internal validity. To the extent that participants are able to complete these activities may help determine resistant starches adherence rates, interpret stool sample analyses, identify treatment safety concerns, and assess whether observed outcomes are attributable to the intervention rather than non-adherence to prescribed medications. Interpretation of pilot trial findings can, therefore, be aided by understanding the extent to which fidelity to the trial intervention (i.e., adherence to resistant starches or placebo), data collection (i.e., stool sample collection, keeping a symptom and dose diary), and adherence to standard care (i.e., prescribed medications) is upheld.

The trial-related activities of interest (i.e., taking resistant starches, collecting stool samples, keeping a diary, adherence to standard care medication) require newly diagnosed patients and their caregivers to change their routines and introduce new behaviors. The National Institute of Health guidelines have conceptualized fidelity to health behavior interventions as encompassing three main components: *delivery* (whether the treatment was delivered as intended), *receipt* (whether participants understand and have the skills and ability to use the treatment), and *enactment* (the extent to which participants apply the treatment in their daily life) [14]. For this study, we are focusing on assessing factors that may be facilitating or impeding the *receipt* of information by patients and caregivers, and participant *enactment* of the four mentioned activities [14]. Understanding barriers and enablers to the receipt and enactment of the aforementioned activities at the pilot stage may help identify opportunities to facilitate fidelity in future multicenter trials.

#### Patient-related barriers and enablers to multiple trial activities

Children and youth who have been diagnosed with IBD, and their caregivers, are often highly motivated to make the necessary changes to their routine to accommodate dietary and treatment needs [12]. However, they may experience challenges to completing activities that are viewed as part of research in addition to their usual medical care [15]. For example, pediatric IBD patients and their caregivers have reported many barriers to medication adherence in medical care settings, including forgetting, being away from home, difficulty swallowing, pain with injectable treatments, interference with other activities, side effects, lack of time, lack of motivation to continue long-term treatments when symptoms improve, inadequate planning, anxiety and depressive symptoms, embarrassment, regiment complexity, and not understanding the reason for taking supplements [16–25]. Barriers to medication adherence contribute to significant non-adherence rates in observational studies ranging between 2 and 93% for children and youth diagnosed with IBD, while frequency rates for missed doses range from 3-57% [16]. Other studies have found 40-50% non-adherence rates based on pill counts [26] and 56-68% non-adherence rates for daily supplements [17]. In adult samples, adherence rates have been shown to vary according to specific medications and to be lower for maintenance regimens [27]. While these rates reflect “real-world” data, non-adherence (and barriers/enablers to adherence) within pediatric IBD trials remains relatively unexplored.

Considerably less is known about barriers to stool sample collection. One study identified lack of information, hygiene concerns, and embarrassment as key barriers to stool sample collection [28]. To our knowledge, there is no research documenting barriers and enablers to keeping a symptom and dose diary.

#### Personal projects analysis

Personal projects analysis (PPA) is a tool that can be used to understand how patients and their families approach integrating trial activities into their daily lives and how they manage competing demands and other priorities that may prevent them, even if unintentionally, from completing trial tasks [29, 30]. Personal projects can be “regarded as a set of interrelated acts extending over time” (p. 276) that an individual chooses to pursue based on a variety of factors, including how much a personal project reflects core values, how it compares to other personal projects, and how complex a personal project is perceived to be [29]. Delving into how multiple trial activities are viewed in relation to other personal projects has the potential to elucidate how and why some participants prioritize research activities and others do not. This approach may shed light on the meaning attributed to engaging in trial activities and how engaging in multiple trial activities is negotiated when these activities conflict with other high priority personal projects.

PPA has previously been used to better understand how other daily activities may help to promote physical activity, patient experiences with adhering to cardiac rehabilitation post myocardial infarction, barriers to goal pursuit among women undergoing treatment for breast cancer, and how adolescents and adults have adapted to various health conditions (e.g., back pain, stroke, diabetes) [31–37]. The strength of this approach lies in emphasizing the cognitive, affective, and behavioral aspects of goal pursuit within the context of a person’s quotidian life. Doing so has generated insights into how other personal projects may conflict or support health behaviors. We aim to use PPA as a tool to understand how participants of a pilot IBD trial integrate trial-related activities (i.e., adhering to standard care medication, daily ingestion of resistant starches, stool sample collection, and keeping a symptom diary) into their daily lives and how they maintain these health behaviors over time, particularly in the presence and absence of barriers and enablers. By doing so, we aim to better understand how engaging in multiple trial activities may impact intervention and data collection fidelity to then identify ways of optimizing fidelity in future trials.

## Methods

### Study objectives

Our primary objective is to assess fidelity considerations alongside the resistant starches pilot trial to better understand the factors associated with adherence to multiple trial activities. From the perspective of patients and caregivers who participate in the pilot trial, we aim to assess the barriers and enablers to the receipt and enactment of trial activities by (1) exploring patient and caregiver understandings of trial activities (i.e., ingestion of resistant starches or placebo, collecting stool samples, keeping a symptom and dose diary, adherence to standard care medication) and instructions for completing them, (2) identifying barriers and enablers to completing trial activities over time, (3) understanding how patients and families incorporate trial activities into their daily lives, and (4) gaining insights into how patients and families address and overcome barriers to completing trial tasks over time.

### Design

To assess fidelity related to receipt and enactment, we will conduct semi-structured interviews with the children, youth, and caregivers that participate in the resistant starches pilot trial. Semi-structured interviews provide participants with an opportunity to reflect on fidelity challenges in greater depth and to consider how engaging in multiple trial activities may have impacted their ability to complete specific trial tasks or to maintain a new medication regimen. To ensure we gain an in-depth understanding of the context within which trial participant lives are situated, we will use PPA as a theoretical framework that facilitates an in-depth exploration of what happens when trial participants return home and strive to complete trial activities amidst the complexities of daily living.

### Setting

The resistant starches pilot trial is being conducted by clinicians at a pediatric IBD clinic based in a large pediatric teaching hospital in an urban setting in Canada. The clinic offers specialized care and support for children and youth living with IBD through the provision of medicines, testing, and follow-up medical team care with access to allied healthcare services (e.g., registered dietitians, social worker, pharmacy assistant). The clinic adopts a learning health system model to clinical research that integrates research activities with standard clinical care practices [38]. A critical component of the learning health system model involves meaningful engagement with patients and caregivers through sharing research results, patient-centered learning events, and educational tools that welcome questions, curiosity, and collaboration from patients and their caregivers [38, 39]. Clinicians associated with this clinic have used this model to conduct preclinical and clinical research aimed at understanding the initiation and progression of IBD and its impact on the wellness of children and youth. The resistant starches pilot trial is a continuation of this larger research program that is embedded within this model.

### Participants

Trial participants between the ages of 8 and 17 years, and their caregivers, who have had the opportunity to take resistant starches (or a placebo) for at least 1 month will be approached for participation in this study.

### Recruitment

The resistant starches pilot trial coordinator who will be acquainted with trial participants will identify and approach prospective patient and caregiver participants either in-person, over the phone, or by email, based on their enrollment in the pilot trial (purposive sampling). Those who express interest in participating in an interview will be directed to a researcher independent of the clinical setting. All interview participants will be recruited from the same site.

### Interview procedure and data collection

#### Researcher as instrument

GC, a clinical research coordinator with a Masters of Arts in social psychology and qualitative methods will conduct all interviews. She will receive guidance from JP, MH, and DM who are experts in health psychology, adolescent health, and pediatric IBD, respectively.

#### Interview guide development

Given the range in age of trial participants, caregivers will be invited to participate alongside their child, or separately, depending on the age of the child and the child’s preferences. Though some have suggested that interviewing children and caregivers separately may encourage greater participation from children, others have noted that younger children may feel more comfortable with a caregiver present and that a caregiver presence may facilitate the interview process through prompts and scaffolding [40–43]. Two interview guides were developed to reflect age-appropriate language and content and one interview guide was developed for use with caregivers (see Additional files 1, 2 and 3). The caregiver interview guide has been developed for use on its own or in tandem with children/youth guides. Interview guides have, therefore, been designed to be flexible and allow the interviewer to adapt the guide based on who is present during the interview (i.e., caregivers). When appropriate, participants will be provided with interview questions ahead of time (e.g., if a caregiver requests to see questions that will be asked of their child).

Trial participant and caregiver interview guides include questions and prompts to identify potential barriers and enablers to the receipt of trial information and the enactment of multiple trial activities, namely, medication adherence, daily ingestion of resistant starches, stool sample collection, and keeping a symptom/dose diary. Interview guides also include questions designed to explore how trial participants and their caregivers addressed barriers, managed competing demands, and shifted their priorities over time. Interview guides will be piloted within the research team and with children and caregivers to ensure questions are clear, relevant, and age appropriate. The content and wording of interview guides will be adjusted based on the acquired feedback. In keeping with an iterative process, guides will continue to be adapted based on participant responses.

#### Interview procedure

As part of the pilot trial, participants are asked to take resistant starches or a placebo for 6 months. Trial participants and caregivers will be recruited after they have completed the intervention period (i.e., after 6 months of ingesting starches/placebo or at the time of withdrawal, whichever comes first). We deemed this time period as optimal for assessing fidelity barriers and enablers as trial participants would have engaged in most trial activities and would be able to speak to challenges in maintaining these trial activities over time, if any arise. Participants who withdraw from the trial before the 6-month visit will be approached within the first month of their request to withdraw. Every effort will be made to conduct interviews within 1 month of when participants withdraw from the trial.

Interested participants will be invited to participate in a one-time interview in person, by phone, or by online video conference. Interviews are expected to last between 30 and 60 min and will be digitally recorded. The interviewer will take notes after the interview to capture initial thoughts, impressions, and noteworthy aspects of the interview. Interview transcripts will not be returned to participants unless specifically requested. Participant checking will not be conducted to minimize the burden of participation on trial participants and their caregivers.

#### Demographic questionnaire for trial participants

Trial participants and caregivers will be asked to complete a demographics questionnaire (see Additional files 4 and 5). The questionnaire includes items to record age, gender, racial or ethnic background, income, education, and family configurations (e.g., number of children in household). These demographic data are needed to describe the participant sample and draw comparisons with the larger pediatric IBD population. Moreover, it is possible that socioeconomic factors may impact trial participants’ access to resources which may, in turn, impact their ability to fulfill trial activities as instructed. Collecting this information may help identify any differences that may arise based on demographic groupings.

### Sampling

The question of predetermined sample size targets in qualitative research is often contentious and dependent on the philosophical assumptions guiding the research process, characteristics of the study (e.g., scope of project, interview structure, participant expertise), and how well the data answer the research question [44–47]. Given that trial participants are a more heterogeneous group (e.g., disease subtype and phenotype, age, symptoms, socioeconomic factors) and that their resistant starches trial experiences are likely to differ, we expect needing to collect between 15 and 30 interviews to adequately answer our research questions and ensure diverse experiences are well represented [47].

### Planned analysis

Interviews will be analyzed using a conventional content analysis where categories describing participant experiences will be inductively generated from participant data [48]. Conventional content analysis will be used to specifically explore trial participant experiences participating in research, engaging with specific trial-related activities, and how participants managed competing demands in relation to adhering to the intervention and data collection trial activities [29]. The emerging coding framework will then be compared to factors associated with personal projects analysis to gain further insights into how fidelity barriers may change over time (i.e., once participants have resumed a routine).

To verify the emerging analyses, a second analyst will review a preliminary set of codes and categories to assess how well the data are represented. Where differences in interpretation arise, the two analysts will discuss to ensure all interpretations are considered until arriving at one that best accounts for participant views and experiences. Data will be analyzed using NVivo 11, a standard qualitative software program.

### Ethics and study progress

The described fidelity study received funding from the Genome Canada 2017 Large-Scale Applied Research Microbiome-Based Precision Medicine in Inflammatory Bowel Disease grant and ethics approval from the Children’s Hospital of Eastern Ontario Research Ethics Board (ID # 20/65X). Data collection is expected to commence in February of 2021 and the results are expected to be submitted for publication by December 2021.

## Discussion

Our process evaluation seeks to identify barriers and enablers to fidelity to multiple trial activities within the context of a pilot trial of resistant starches for IBD. We seek to assess fidelity barriers and enablers to the receipt and enactment of four activities—daily ingestion of resistant starches, collection of stool samples, keeping a symptom and dose diary, and adhering to standard care medications. To do so, we will conduct interviews with patients and caregivers to assess how well trial activity instructions are received, and perhaps more importantly, how trial participants and their caregivers proceeded to enact and integrate these activities into their daily lives.

Two elements of the proposed fidelity study are unique and potentially of interest to others conducting pilot and feasibility studies. First, identifying barriers and enablers to the trial intervention and data collection fidelity at the pilot trial phase can serve to inform the conduct of future multisite trials. Seeking to assess fidelity issues at the outset allows for the exploration of how the context in which a pilot study is conducted supports or impedes fidelity. This, in turn, may aide in identifying what challenges may be expected and addressed prior to trial conduct in other settings.

Second, we have opted to deepen our understanding of the enactment stage of fidelity by using personal projects analysis to explore how trial participants engage with trial activities once they have left the clinic and return to their daily routines. Using a theoretical tool that enables in-depth exploration of goal conflicts and goal pursuits will help generate insights regarding what differentiates those who manage high fidelity to multiple trial activities and those who are prevented from completing tasks as prescribed.

Assessing fidelity at the pilot phase of a clinical trial presents opportunities to improve both the interpretation of pilot trial outcomes and the design and conduct of future multisite trials. Using theory and a fidelity framework that emphasizes receipt and enactment as component parts further creates opportunities to expand our conceptualizations of fidelity and demonstrates how theory and frameworks can be used to optimize trial design. We, thereby, aim to contribute to the growing field of process evaluations by providing an example of how theoretically informed fidelity assessments can be conducted alongside pilot trials to inform future multisite trials.

## Supplementary Information

**Additional file 1.** Caregiver Interview Guide.

**Additional file 2.** Youth Interview Guide.

**Additional file 3.** Child Interview Guide.

**Additional file 4.** Demographics Questionnaire – Parent.

**Additional file 5.** Demographics Questionnaire – Child/Youth.

## Data Availability

The datasets generated and/or analyzed during the current study will not be made publicly available in order to protect the privacy and confidentiality of study participants. However, de-identified, aggregated data may be made available from the corresponding author on reasonable request.

## Abbreviations

IBD: Inflammatory bowel disease
PPA: Personal projects analysis
