# Supplementary material for: Factors contributing to fidelity in a pilot trial of individualized resistant starches for pediatric inflammatory bowel disease: a fidelity study protocol
Source: Pilot Feasibility Stud. 2021 Mar 19;7:75. doi: 10.1186/s40814-021-00815-1 (PMC7976693; doi:10.1186/s40814-021-00815-1)
Supplement: Supplementary file 1 — Additional file 1. Caregiver Interview Guide. [file 40814_2021_815_MOESM1_ESM.docx]

Appendix B

**Caregiver Interview Guide**

**Introduction to the study**

Thank you for taking the time to speak with me today. I’m a research coordinator at the Ottawa Hospital Research Institute working with Dr. Presseau, a health psychologist and scientist who also works at the Ottawa Hospital Research Institute. We’ve been working with Dr. Mack and the staff at the CHEO IBD Centre on a study that is looking at how people manage when they’re asked to do several things as part of a clinical trial.

Specifically, we’re interested in your experiences participating in the resistant starches study. We would like to know more about how you managed with helping your child do things like take their IBD medications, keep up with taking the resistant starches, collect stool samples, and keep a symptom/dose diary on top of everything else you do as a caregiver. We know it can be challenging so we’d like to know more about how it went for you. As part of this interview study, we are speaking with people who have participated in the resistant starches pilot trial, like yourself, to get a sense of what the experience was like.

We’re hoping to learn from you about what helped you and what was challenging about participating in the trial so that we can better prepare for future trials that may be done at other IBD centres. In other words, our goal is to learn more about the ways we can better support families who choose to participate in these types of trials.

Did you have any questions or concerns about what we just covered?

**Participant rights and consent**

Okay great, now I just want to emphasize some key points that were explained in the consent form. First, please know that your participation is totally voluntary. That means that at any point during the interview you can decide you no longer want to participate or that you do not want to answer any questions. I want you to also know that whether you choose to participate or not will not affect the care you receive at the CHEO IBD Centre.

The interview will take about 30-60 minutes, depending on how much you would like to share, and as a thank you for participating, you and your child will get a $25 gift card (one per family).

You should know I will record our interview today to make sure we get an accurate account of what you share and so that we can type it up later. Once we have the interview typed up we will take out any mentions of places, names or unique details that may make your identity known to others. Your privacy is important to us so we will do everything in our power to keep our interview confidential. That means that only the members of the research team who are based at the OHRI will have access to things like the interview audio recordings and transcripts. Members of the research team who are based part of the CHEO IBD clinic won’t have access to the interview audio or transcript, but will see summaries of the results including quotes from your interview.

So, to further protect your identity, we will ask that you provide us with a pretend or fake name that we can use with your quotes. What fake name would you like to use? And what pronoun would you like to use with that name?

Fake name: _________________________ Pronoun: _______________________

Any questions about anything we’ve covered so far?

Okay great. Please know **there are no right or wrong answers**, I’m really just interested in hearing about what you think. Also, please keep in mind that I am **not a clinician,** my background is in social psychology, so I may ask for clarifications from time to time.

Any questions before we start?

**[begin recording**]

May I get your **consent** to proceed with the interview **on the record**?

**Part 1 – Introduction to Trial**

I’d love to know more about what participating in that study has been like for you.

1. Thinking back, how did you first hear about the resistant starches study?
   1. How was it introduced to you? By whom?
2. What made you decide to participate in the resistant starches pilot trial?

**Part 2 –** **Experiences with Trial Activities**

Now I’d like to know a bit more about your experiences with some of the specific activities you and your child were asked to do as part of the trial. I’m interested in hearing about what it was like keeping up with your child’s prescribed IBD medications, helping them take their daily resistant starch dose, collecting stool samples, and keeping a symptom diary.

1. What was your role in helping your child do each of these four activities?
   1. How did you help/support your child, if at all?
   2. What was your child responsible for doing?
   3. Who else was involved in helping your child complete these four tasks?
2. What resources or skills training did you receive, if any, to help you be part of the resistant starches study?
   1. Note: if mention brochure, ask about experiences using it
3. Tell me about your experiences supporting your child to take their prescribed medications.
   1. What were the instructions you and your child received for taking the medications?
      1. probe for different meds, doses, timing, complexity
   2. What did you think of the medication regimen? How did it go?
   3. Tell me about a time when they did not take their prescribed medications.
      1. What happened? What got in the way?
   4. What was the most difficult part about helping your child take their medications?
4. Tell me about your experiences helping your child with taking the resistant starches on a daily basis.
   1. What were the instructions you were given for helping your child take the resistant starches?
   2. Tell me about a time when your child did not take the resistant starches.
      1. What happened?
      2. What got in the way?
   3. What was the most difficult part about helping your child take the resistant starches?
5. Tell me about your experiences with collecting the stool samples.
   1. What instructions were given to you for helping your child collect stool samples?
   2. Tell me about a time when you/your child were supposed to collect a sample and did not collect one.
      1. What happened? What got in the way?
   3. What was the most difficult part about helping your child collect the stool samples?
      1. How did you manage when dealing with those challenges?
6. Tell me about your experiences keeping track of your child’s RS doses and symptoms.
   1. What instructions were you given for helping your child keep track of their doses and symptoms?
   2. What was your experience like using the dose and symptom tracking pages of the brochure?
   3. What worked well, what did not work so well?
   4. Tell me about at time when entries were not recorded.
      1. What happened? What got in the way?
   5. What was the most difficult part about helping your child keep track of their doses and symptoms?

**Part 4 – Managing Competing Demands**

Now I’m interested in hearing more about how you managed to help your child with these activities along with everything else you do as a caregiver.

1. What was it like for you to help your child stay on top of the four activities we’ve discussed?
2. What was it like making the trial activities part of your routine?
   1. What strategies or tools did you use?
   2. What was challenging? What was easy?
3. Thinking back over the past few months, what else was going on in your life at the time that made it challenging to do the activities we’ve been talking about?
   1. How did you manage to continue helping your child with the study tasks while dealing with all the demands on your time?
   2. How did these competing demands affect your ability to support your child with the activities we’ve been talking about?
4. [If withdrew from trial] Tell me more about what was happening when you withdrew from the trial.
   1. What led you to withdraw?

**Part 5 – Reflecting on Trial Experiences**

1. Thinking back to what was going on at the time, what would have been helpful to you to make participating in the trial easier?
   1. What would have made any of the four activities we’ve talked about, easier to do?

What information/resources would have been helpful?

- 1. What kind of social supports would have been helpful?

1. How would you describe your overall experience helping your child participate in the trial so far?
2. What advice would you give to other people who are considering participating in a trial like this?

Is there anything else you’d like to share with me today?

Thank you so much for taking the time to speak with me!
