# Supplementary material for: Factors contributing to fidelity in a pilot trial of individualized resistant starches for pediatric inflammatory bowel disease: a fidelity study protocol
Source: Pilot Feasibility Stud. 2021 Mar 19;7:75. doi: 10.1186/s40814-021-00815-1 (PMC7976693; doi:10.1186/s40814-021-00815-1)
Supplement: Supplementary file 2 — Additional file 2. Youth Interview Guide. [file 40814_2021_815_MOESM2_ESM.docx]

Appendix D

**Youth Interview Guide**

**Introduction to the study**

Thank you for taking the time to speak with me today. I’m a research coordinator working with Dr. Presseau, a scientist at the Ottawa Hospital Research Institute. We’ve been working with Dr. Mack and the staff at the CHEO IBD Clinic on a study that is looking at how people manage when they’re asked to do many things as part of a research study. Specifically, we’re interested in hearing what it was like for you to participate in the resistant starches study. We would like to know more about what it was like taking your IBD medications, taking the resistant starch powder, collecting stool samples, and keeping track of your doses and symptoms using the study brochure. We know it can be challenging to do all those things on top of everything else you do so we’d like to know more about how it went for you. As part of this study, we are speaking with people who have participated in the resistant starches study, like yourself, to get a sense of what the experience was like.

We’re hoping to learn from you about what helped you and what was hard about participating in the trial so that we can better prepare for other research studies that may be done at other IBD centres. In other words, we want to know what we can do to make it easier to participate in research studies like the resistant starches trial.

Did you have any questions about anything I’ve said so far?

**Participant rights and consent**

Okay great, now I just want to go over some key points that were talked about in the consent form. First, please know that your participation is totally voluntary. That means that at any point during the interview you can decide you no longer want to participate or that you do not want to answer any questions. I want you to also know that whether you choose to participate or not will not affect the medical care you get at the CHEO IBD clinic.

The interview will take about 30-60 minutes, depending on how much you would like to share, and as a thank you for participating, you and your caregiver (one per family) will get a $25 gift card to Chapters.

You should know I will record our interview today so that we can type it up later. Once we have the interview typed up we will take out any mentions of places, names or specific details that may make your identity known to others. Your privacy is important to us so we will do everything in our power to keep our interview confidential. That means that only the members of the research team who are based at the OHRI will be able to listen to your interview recording or see your transcript. People who are part of the research team who work at the CHEO IBD clinic won’t be able to listen to your interview recording or see your transcript, but they will see summaries of the results including quotes from your interview.

So, to better protect your identity, we will ask that you provide us with a fake name that we can use with your quotes. What fake name would you like to use?

Fake name: _________________________

What pronoun (e.g., he, she, they) would you like to use with that name?

Pronoun: ___________________

Any questions about anything we’ve covered so far?

Okay great. Please know **there are no right or wrong answers**, I’m really just interested in hearing about what you think. Also, please keep in mind that I am **not a clinician,** so I may have to ask you to explain things to me that I am not familiar with.

Any questions before we start?

**[begin recording**]

May I get your **consent** to proceed with the interview **on the record**?

**Part 1 – Trial initiation**

So, I hear you have been participating in the resistant starches study.

1. How did you first hear about the resistant starches study?
   1. What did they tell you about it?
2. What made you decide to participate in the resistant starches study?
3. Can you tell me a bit about, what’s it been like to be part of the study for the past few months?
4. What were you asked to do as part of this study?
   1. Probe for meds, RS, poop, diary
5. What kind of instructions did you get? / How did you know what you were supposed to do?
   1. [use visual aids – e.g., how did you know what you were supposed to do with this page of the brochure?]
   2. Was there anything that you were taught to do so you could be part of the RS study?

**Part 2 – Trial activities and managing competing demands**

So, I’m interested in hearing more about what it was like to take your IBD medications, take the RS powder, get your poop samples, and use the tracking pages in the brochure.

1. Thinking back over the last 6 months, what has it been like taking your IBD meds/resistant starches/collecting stool samples/keeping diary?
   1. What went well?
   2. What did not go so well?
   3. How much work was it for you to do these activities?
2. How did you make these activities part of your daily routine?
   1. Tell me about it, what was your routine?
   2. What tricks and strategies did you use to stay on top of things?
      1. Probe for reminders, tools, social support, habit
3. Which of these activities (meds, powder, poop, diary) did you find the hardest to do? What made it hard?
   1. What happened when you ran into problems?
   2. Was there anyone who helped you when you ran into problems with ____ [meds, RS, poop, diary]
4. Which was the easiest of the four activities (meds, powder, poop, diary) to do? What made it easy?
5. Tell me about a time when you weren’t able to do one of the activities (you pick which). What happened?

Now I’m interested in hearing more about how you managed to do these activities along with everything else you do (e.g., school, chores, fun).

1. Sometimes, life gets pretty busy. Tell me about a time when being busy made it hard to do any of the four activities (meds, powder, poop, diary) we’ve been talking about.
   1. What made your life so busy?
   2. How did you manage (or not) to continue doing the activities we’ve been talking about?
   3. What was most important to you during that time?
2. [If withdrew from trial] Tell me more about what was happening when you withdrew from the trial.
   1. What led you to withdraw?

**Part 3 – Reflecting on Trial Experiences**

1. Thinking back to what was going on at the time, what would have been helpful to you to make participating in the trial easier?
   1. What would have made any of the four activities we’ve talked about, easier to do?
   2. What material resources would have been helpful?
   3. What information would you have liked to have had?

What kind of social supports would have been helpful?

1. How would you describe your overall experience participating in the trial so far?
   1. What has gone not so well/could be better?
2. What tips or advice would you give to other young people who are considering participating in a trial like this?

Is there anything else you’d like to share with me today?

Thank you so much for taking the time to speak with me!
