# Supplementary material for: Factors contributing to fidelity in a pilot trial of individualized resistant starches for pediatric inflammatory bowel disease: a fidelity study protocol
Source: Pilot Feasibility Stud. 2021 Mar 19;7:75. doi: 10.1186/s40814-021-00815-1 (PMC7976693; doi:10.1186/s40814-021-00815-1)
Supplement: Supplementary file 3 — Additional file 3. Child Interview Guide. [file 40814_2021_815_MOESM3_ESM.docx]

Appendix C

**Child Interview Guide**

**Introduction to the study**

Thank you for taking the time to talk to me today. I work at the Ottawa Hospital with a scientist Dr. Presseau. We’ve been working with Dr. Mack and the people at the CHEO IBD Clinic to look at what kids like you think about being in the resistant starches study. We would like to know more about what it was like to take your medications, to eat the resistant starches powder, to collecting your poop in little jars, and to keep track of your symptoms, or what your body was feeling, in a diary. I know it can sometimes be hard to do all those things so I’d like to know more about how it was to you. I’m also talking to other kids and families that participated in the same study you did.

I’m hoping to learn from you about what helped you and what was hard about being in the study so that we can make it even easier for other kids to participate in studies like this.

Did you have any questions about anything I’ve said so far?

**Participant rights and consent**

Okay great, now I just want to go over some important points that we talked about before. First, the decision about whether you want to talk to me is totally up to you! You don’t have to if you don’t want to, no one will be mad at you or sad if you decide you don’t want to talk to me. You can also decide you don’t want to talk anymore at any time, even after we’ve started, you just let me know. You also don’t have to answer questions you don’t want to answer – if anything makes you uncomfortable just say ‘I don’t want to answer that one’.

The interview will take about 30 minutes but we can make it shorter or longer, totally up to you.

So, we want to keep your name secret so that it’s hard for people to know what you said. What fake name would you like to use? You can call yourself anything you want!

Fake name: _________________________

Would you like [fake name] to be called a “he, she, they” or something else?

Pronoun: ____________________

Any questions for me so far?

Okay great. Please know **there are no right or wrong answers**, I’m really just interested in what you think.

Any questions before we start?

**[begin recording**]

Do you agree to participate in this study with me today?

1. So, I heard you have been taking part in a study about resistant starches (*use appropriate language based on staff and caregiver input*). Can you tell me about that?
2. What were you asked to do as part of that study?

I’m interested in hearing more about what it was like to take medicine, eat the powder that comes in packets, collect your poop in jars, and keep track of how your body’s feeling (symptoms) in a diary.

1. Which of those did you have to do? Which of those were you responsible for?
   1. Did anyone help you do any of those activities?
2. What is it like to take the medicine your stomach doctor gave you?
   1. How easy or hard was it to take your medicine?
   2. Tell me about a time when you did not take your medicine (e.g., was there a time you forgot?).
      1. What happened?
      2. What got in the way?
3. I’ve never drank or eaten the powder, can you tell me what is it like to eat/drink the powder that comes in packets?
   1. How easy or hard was it to eat the powder?
   2. Tell me about a time when you did not eat the powder.
      1. What happened?
      2. What got in the way?
4. What was it like to put your poop in the little jars?
   1. How many times did you collect poop in jars?
   2. Tell me about a time when you were going to collect poop in jars but you did not.
      1. What happened?
      2. What got in the way?
   3. What was the hardest part about collecting poop in jars?
5. What was it like to use the study booklet? [show visual]
   1. What was it like to use page 14 [show visual] to keep track of how your body was feeling?
   2. What was it like to use pages 12 and 13 to keep track of when you ate the powder?
   3. How did you know what you were supposed to do with these pages in the brochure?
   4. Tell me about at time when you were not able to use these pages. Tell me about a time when you did not write down how you were feeling?
      1. What happened?
      2. What got in the way?
   5. Tell me about a time when you were not able to write down when you ate the powder (i.e., using pages 12 and 13).
      1. What happened? What got in the way?
   6. What was the hardest part about using the brochure? What was the easiest part?
6. Sometimes, you have a busy day doing lots of stuff. Can you tell me about some of the things you do? What did you do yesterday?
   1. What made your life so busy?
   2. Was it ever so busy that you it was hard to do any of the activities we’ve been talking about?
      1. What happened?
      2. What did you do to help you do the activities even though it was so busy?
      3. What happened when you were not able to do the activities?
7. [If withdrew from trial] Tell me more about what was happening when you decided to stop being part of the resistant starches study (i.e., taking the powder).
   1. What made you and your family decide to stop being part of the study?

**Part 5 – Reflecting on Trial Experiences**

1. Thinking about the activities we talked about, taking your medicine, eating or drinking the powder, putting your poop in jars, or using the study booklet…
   1. What would have made it easier to take your medicine?
   2. What would have made it easier to eat/drink the powder?
   3. What would have made it easier to put your poop in jars?
   4. What would have made it easier to use the study booklet?
2. What would you tell other kids who are about to start participating in the study?
   1. What would you want to tell them to help them get ready?

Is there anything else you’d like to share with me today?

Thank you so much for taking the time to speak with me!
