# Supplementary material for: Factors contributing to fidelity in a pilot trial of individualized resistant starches for pediatric inflammatory bowel disease: a fidelity study protocol
Source: Pilot Feasibility Stud. 2021 Mar 19;7:75. doi: 10.1186/s40814-021-00815-1 (PMC7976693; doi:10.1186/s40814-021-00815-1)
Supplement: Supplementary file 4 — Additional file 4. Demographics Questionnaire – Parent. [file 40814_2021_815_MOESM4_ESM.docx]

Appendix D

**Demographics Questionnaire – Parent**

Thank you for taking a moment to complete this questionnaire. The questions below ask about your background (e.g., age, gender, education, income). It is important for us to gather this information to better describe who we speak with and to better understand whether there are differences or similarities in experiences that are associated with things like gender, family size, or income. Please note that individual demographic information will not be reported. Instead, information will be presented as a summary of several or all responses. We recognize these questions are personal, so please take a moment to consider each one. If you are uncomfortable responding to any of these questions, please feel free to leave them blank.

1. Your relationship to the child/youth who participated in the resistant starches pilot trial:
   1. Mother
   2. Father
   3. Grandparent
   4. Aunt/Uncle
   5. Other (please specify): _____________
2. What year were you born?
3. What year was your child (resistant starches trial participant) born?
4. What is your gender? __________
5. What is your child’s current gender? ________
6. What is your country of birth? ____________
7. What is your child’s country of birth? __________
8. What are the ethnic and/or cultural origins of you and your ancestors? ______________________
9. What is your marital status?
   1. Never married
   2. Married
   3. Common-law or domestic partnership
   4. Divorced
   5. Separated
   6. Widowed
   7. Prefer not to answer
   8. Other
10. How many children do you have? _____________
11. How many people currently live with you, including children? __________
12. What is the highest level of education you have completed?
    1. Some elementary school
    2. Finished elementary school
    3. Some high school
    4. Finished high school or equivalency certificate
    5. Certificate of apprenticeship or other trades certificate/diploma
    6. Some college/university
    7. Finished college/university
    8. Some graduate studies (e.g., MA, MBA, MSc, PhD)
    9. Finished graduate degree
    10. Prefer not to Answer
    11. Other
13. What is your employment status?
    1. I work full-time.
    2. I work part-time.
    3. I am on sick leave.
    4. I am on disability.
    5. I am looking for work.
    6. I do not work.
    7. I am retired.
    8. Prefer not to answer
    9. Other
14. Which of the following best represents your total household income before taxes in 2019?
    1. Under $45,000
    2. $45,001- $95,000
    3. $95,001- $145,000
    4. $145,001-$210,000
    5. Over $210,000
    6. Prefer not to answer
    7. Other
15. Which treatment group do you believe your child was assigned to?
    1. Placebo group
    2. Resistant starches group
    3. I don’t know
    4. Prefer not to say
