# Supplementary material for: Factors contributing to fidelity in a pilot trial of individualized resistant starches for pediatric inflammatory bowel disease: a fidelity study protocol
Source: Pilot Feasibility Stud. 2021 Mar 19;7:75. doi: 10.1186/s40814-021-00815-1 (PMC7976693; doi:10.1186/s40814-021-00815-1)
Supplement: Supplementary file 5 — Additional file 5. Demographics Questionnaire – Child/Youth. [file 40814_2021_815_MOESM5_ESM.docx]

Appendix E

**Demographics Questionnaire – Child/Youth**

Thank you for taking a moment to fill out this form! The following questions ask about you (e.g., age, gender). We ask these questions to everyone who is taking part in the interview study so we can better describe who we speak with and to see if there are any differences or similarities based on things like age and gender. We won’t share anything about you specifically. Instead, we will put lots of responses together so no one can see what you said.

1. How old are you?
2. What grade are you in?
3. What is your gender? (e.g., I am a girl, I’m not sure) _________
4. What is your ethnic and/or cultural background?

_______________________________

1. How many brothers and sisters do you have? ______________
2. How many people currently live in your home with you, including kids? _____________
3. Which treatment group do you believe you were assigned to?
   1. Placebo group
   2. Resistant starches group
   3. I don’t know
   4. Prefer not to say
